# Priming for Improved Hand Strength in Persons with Chronic Tetraplegia: A Comparison of Priming-Augmented Functional Task Practice, Priming Alone, and Conventional Exercise Training

**DOI:** 10.3389/fneur.2016.00242

**Published:** 2017-01-17

**Authors:** Joyce Gomes-Osman, Jacqueline A. Tibbett, Brandon P. Poe, Edelle C. Field-Fote

**Affiliations:** ^1^Department of Physical Therapy, University of Miami Miller School of Medicine, Coral Gables, FL, USA; ^2^The Miami Project to Cure Paralysis, University of Miami Miller School of Medicine, Miami, FL, USA; ^3^Shepherd Center, Crawford Research Institute, Atlanta, GA, USA; ^4^Division of Physical Therapy, Emory University School of Medicine, Atlanta, GA, USA

**Keywords:** spinal cord injury, activities of daily living, hand function, rehabilitation, human movement system

## Abstract

Many everyday tasks cannot be accomplished without adequate grip strength, and corticomotor drive to the spinal motoneurons is a key determinant of grip strength. In persons with tetraplegia, damage to spinal pathways limits transmission of signals from motor cortex to spinal motoneurons. Corticomotor priming, which increases descending drive, should increase corticospinal transmission through the remaining spinal pathways resulting in increased grip strength. Since the motor and somatosensory cortices share reciprocal connections, corticomotor priming may also have potential to influence somatosensory function. The purpose of this study was to assess changes in grip (precision, power) force and tactile sensation associated with two different corticomotor priming approaches and a conventional training approach and to determine whether baseline values can predict responsiveness to training. Participants with chronic (≥1 year) tetraplegia (*n* = 49) were randomized to one of two corticomotor priming approaches: functional task practice plus peripheral nerve somatosensory stimulation (FTP + PNSS) or PNSS alone, or to conventional exercise training (CET). To assess whether baseline corticospinal excitability (CSE) is predictive of responsiveness to training, in a subset of participants, we assessed pre-intervention CSE of the thenar muscles. Participants were trained 2 h daily, 5 days/week for 4 weeks. Thirty-seven participants completed the study. Following intervention, significant improvements in precision grip force were observed in both the stronger and weaker hand in the FTP + PNSS group (effect size: 0.51, *p* = 0.04 and 0.54, *p* = 0.03, respectively), and significant improvements in weak hand precision grip force were associated with both PNSS and CET (effect size: 0.54, *p* = 0.03 and 0.75, *p* = 0.02, respectively). No significant changes were observed in power grip force or somatosensory scores in any group. Across all groups, responsiveness to training as measured by change in weak hand power grip force was correlated with baseline force. Change in precision grip strength was correlated with measures of baseline CSE. These findings indicate that corticomotor priming with FTP + PNSS had the greatest influence on precision grip strength in both the stronger and weaker hand; however, both PNSS and CET were associated with improved precision grip strength in the weaker hand. Responsiveness to training may be associated with baseline CSE.

## Introduction

Injury to the cervical spinal cord results in tetraplegia and associated impairment or loss of upper extremity (UE) control and sensory function. These impairments can result in marked limitations in the ability to perform functional tasks, and severely restrict independence and quality of life. Tetraplegia comprises one half of the approximately 17,000 spinal cord injuries (SCIs) that occur each year in the United States (1). Not surprisingly, this group frequently cites recovery of arm and hand function as the single most important priority in terms of functional restoration (2–4). Yet inpatient therapy for persons with tetraplegia has little if any focus on restoration of hand function (5), despite the fact that in persons with tetraplegia hand muscle force generation is highly correlated with success or failure in the ability to perform common functional tasks (6).

In non-disabled persons, the level of excitability of the motor cortex is the primary determinant of muscle force generation (7). Following tetraplegia damage to the descending tracts limits the amount and rate of transmission of information from the cortex to the spinal cord (8), this impaired transmission of signals through the corticospinal pathways limits the ability to generate hand muscle forces resulting in weak precision grip and power grip strength. For these reasons, corticospinal excitability (CSE) is of great relevance for individuals with tetraplegia, since increasing the ability of the cortex to drive signals through the spared spinal pathways should result in improved ability to activate the spinal motoneurons and be associated with increased strength.

Beyond the relationship between cortical excitability and force generation, in persons with tetraplegia the size of the motor potential evoked during precision grip is correlated with the sensory function in the median nerve distribution (9). The motor and sensory cortices share a reciprocal relationship; the state of excitability of the motor cortex is known to influence the state of excitability of the somatosensory cortex during active exploration (10). Because of the communication between cortical areas, changes in activity of one area lead to changes in functional connectivity (11) that have the potential to change the activity of other cortical areas. It therefore seems plausible that changes in excitability of the motor cortex may be accompanied by parallel changes in excitability of the somatosensory cortex. However, while improvements in sensory function have been observed in persons with SCI following participation in UE training (12), it is not known whether there is a relationship between the changes in hand force production and change in sensory function.

Motor priming is receiving considerable attention as a way of augmenting the effects of rehabilitation-related training in neurologic clinical populations (13). Much of the early work related to motor priming to improve hand function in persons with tetraplegia centered on the use of peripheral nerve somatosensory stimulation (PNSS) (12, 14–17). More recently, there has been a growth in interest related to technology-intensive approaches (18–24); however, the potential of these approaches to have broad clinical impact may be restricted by their limited accessibility. These studies have appropriately focused on priming-related changes in skilled hand function, however, given that priming is intended to increase corticomotor excitability these approaches might also be expected to have an effect on force production.

Numerous studies have shown that skill training is associated with increased CSE (25–27), and skill training thereby represents an ecologically sound approach to priming the motor cortex. Likewise, PNSS is another clinically accessible approach to priming corticomotor activation (28, 29), wherein activation of sensory afferents is used to excite the somatosensory cortex, which in turn drives excitability in the motor cortex (30). In persons with tetraplegia, PNSS combined with skill training in the form of functional task practice (FTP) of hand motor activities is associated with increased precision grip forces (12, 14, 17), and with increased CSE (12, 17).

Conventional UE exercises for persons with tetraplegia typically consist of progressive resistance training and endurance exercises. While conventional exercise is not considered to be an approach to motor priming, in non-disabled individuals, some studies have concluded that resistance training is associated with increased CSE (26, 31), and with greater twitch forces of the cortically evoked motor response (32). This may suggest that changes in CSE are similar for skill training and strength training (26). Conversely, other studies have suggested that resistance training is associated with a decrease in the size of motor-evoked potentials (MEPs) (33, 34), however, the conclusions may depend on the timing of the CSE testing relative to the end of training (34). In persons with tetraplegia, there are surprisingly few studies of progressive resistance training (without concurrent functional electrical stimulation) directed at improving strength in the paretic muscles. In the three available studies in persons with tetraplegia, resistance training has been shown to improve biceps (35), chest (35), and wrist extensor muscle strength (36, 37). While there are no published studies of the influence of resistance training on CSE in persons with tetraplegia, a study of paretic limb resistance training in persons with stroke provides preliminary support for the idea that strength changes in the paretic limb are accompanied by changes in CSE (38).

Evidence indicates that FPT + PNSS and PNSS each has the potential to prime the motor cortex and increase descending volitional drive to the spinal motoneurons, thereby increasing force production. The effects of cortical priming may also extend to the somatosensory cortex and result in improvements in sensory function. The purpose of this study was to assess, in participants with tetraplegia, the multisession effects of two different approaches to priming the corticospinal system (FTP + PNSS, PNSS) compared to conventional exercise training (CET). Outcomes of interest were change in paretic muscle precision grip and power grip strength, and hand sensory function. In addition, we sought to determine whether baseline measures were predictive of responsiveness to training by evaluating the relationship between the baseline and post-intervention values. In a subset of participants, we assessed the influence of baseline levels of CSE on responsiveness to training.

## Materials and Methods

### Participants

Participants were recruited from The Miami Project to Cure Paralysis (TMP) research volunteer database or from direct contact with individuals who visited TMP and requested to participate. All participants gave written and verbal informed consent to participate in the study that had been approved by the Human Studies Research Office at the University of Miami Miller School of Medicine. To be considered for inclusion, participants had to have the following: cervical SCI (traumatic or non-traumatic) with motor level at or above C7 as defined by the International Standards for Neurological Classification of Spinal Cord Injury (ISNCSCI) (39) sustained at least 1 year prior to the onset of participation; American Spinal Injury Association (ASIA) Impairment Scale (AIS) classification A–D; and thenar muscle grade of ≥1/5, i.e., ability to voluntarily generate at least a trace contraction of the thenar muscles. We included persons with neurological classification of A and B (ie, motor-complete) as these individuals may have partial preservation of innervation that extends several segments below the neurological level of injury (39), allowing them to generate volitional contraction of the thenar muscles. Exclusion criteria were as follows: inability to tolerate sitting for 2 h, history of UE tendon transplant(s), history of head injury, or seizures that would preclude CSE testing using transcranial magnetic stimulation (TMS).

Of the entries in the TMP research volunteer database, 372 had sufficiently complete information to indicate that they appeared to meet the inclusion criteria based on a search query that included injury level, AIS classification, and excluded those with history of head injury or use of a ventilator. In addition, monthly updates based on new database entries were added over the course of the study. Individuals were contacted by phone if the database query indicated that they seemed to meet the inclusion criteria. Potential participants who lived locally were asked to visit TMP for screening. Individuals from outside the local area were asked to send a recent ISNCSCI assessment and a video recording of their hands demonstrating their attempts to perform thumb movements.

Prior to baseline testing, neurologic classification was performed according to the ISNCSCI assessment (39) to document neurological level of injury, sensory function, and motor function. Upper extremity motor scores (UEMSs) were acquired for the five key muscle groups of the upper extremities (elbow flexors, wrist extensors, elbow extensors, finger flexors, and finger abductors) (39). The ISNCSCI exam was performed by an examiner who was not otherwise involved in the study.

### Randomization and Blinding

To ensure equivalence of baseline functional ability among intervention groups, participants were allocated to the groups using a stratified random assignment based on a timed test of hand function [Jebsen-Taylor Hand Function Test (40)]. Participants were stratified into one of two functional levels based on total time to complete the seven timed test items (using the hand perceived by the participant to be more functional). Stratification levels were derived from data from our previous studies and were defined as follows: Stratum 1 (lower functioning) = total time of >200 s; Stratum 2 (higher functioning) = total time of <200 s (15). Each of the strata had an associated container holding a sequence of envelopes organized in the order of the randomization. To conceal allocation, the computer-generated randomization and organization of the envelopes were performed by an individual not otherwise involved in the subject allocation or data collection.

### Interventions

Participants engaged in FTP + PNSS, PNSS, or CET in 2-h sessions, 5 days per week for 4 weeks (target = 20 sessions). Missed days were made up so that all participants had at least 17 training sessions.

### Functional Task Practice (FTP)

Functional task practice training of bimanual tasks was performed according to previously published protocols (15–17). The FPT activities were intended to incorporate movements and motor strategies that comprised common everyday activities. FTP activities were carried out while the participant received PNSS (described below).

**Figure 1:**
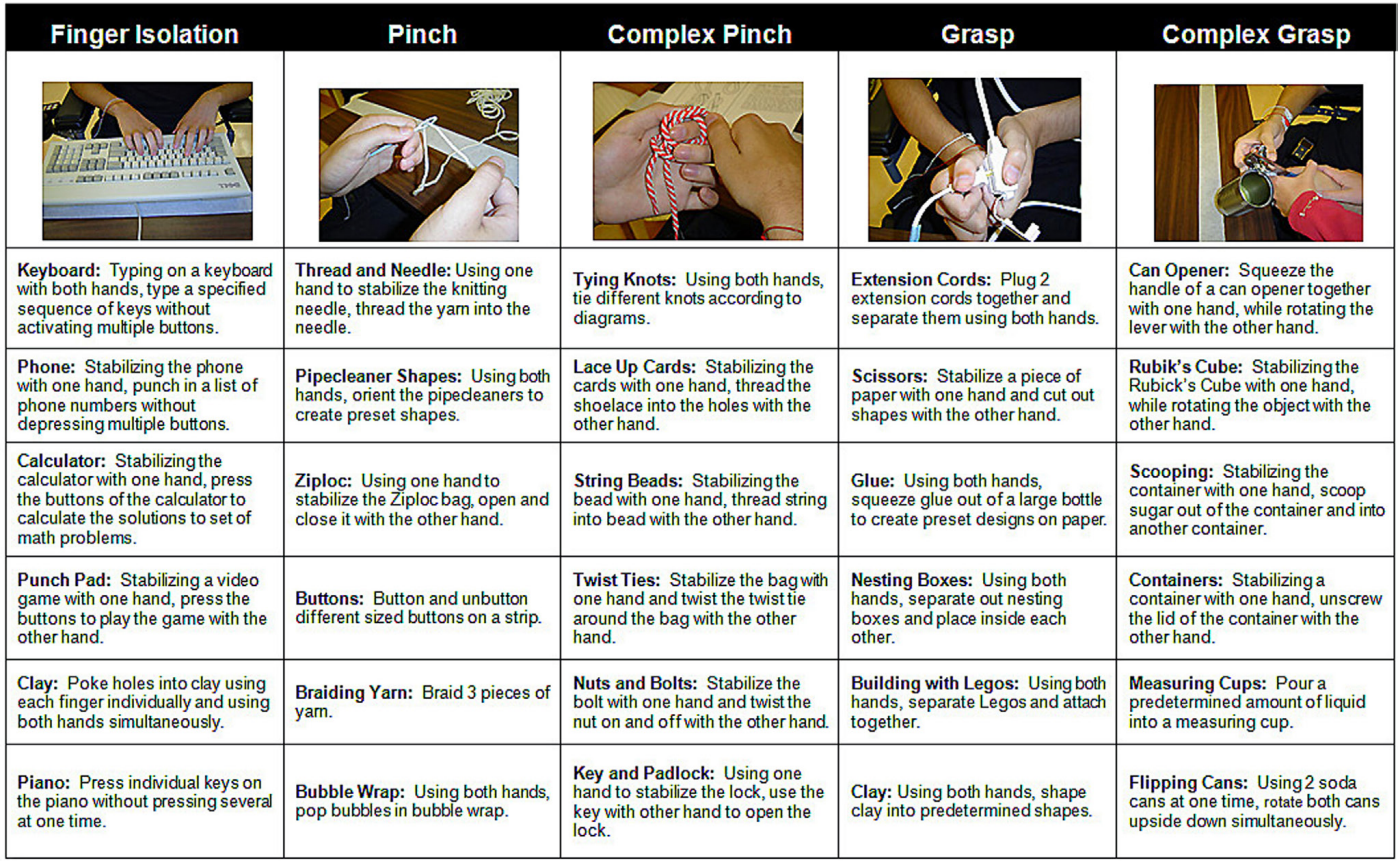
**Sample bimanual functional task practice activities**. Participants engaged in each category of activity for at least 20 min. The therapist provided verbal or tactile cues as needed to limit the use of compensatory strategies.

FTP training comprised structured practice of bimanual tasks that emphasized six categories of activities thought to represent different aspects of functional hand use (15–17). Bimanual activities consisted of both symmetrical (wherein both hands perform similarly, e.g., braiding rope) and asymmetrical tasks (wherein one hand performs the fine motor activity, while the opposite hand stabilized the object being manipulated, e.g., cutting shapes from greeting cards with scissors). Participants were required to spend at least 20 min practicing each of the six, category of activity: independent finger movement, precision grip (pinch), pinch with object manipulation, power grip (grasp) complex power grip (involving object manipulation), finger isolation, and whole arm movement (Figure 1). Precision grip tasks involved both tip-to-tip activities and lateral prehension activities; participants also had the option to use a hand exercise workstation (ReJoyce, Rehabtronics Inc., Edmonton, AB, Canada). In order to increase practice opportunities for the weaker hand, during the practice of asymmetrical tasks the participants were instructed to use the weaker hand to perform the fine motor activity and stabilize the object with the stronger hand. Feedback was given by a therapist to discourage (or decrease) use of compensatory strategies (such a tenodesis grasp to manipulate an object instead of a precision grip). While verbal cuing was the most frequently used form of feedback, in the early days of training the therapist occasionally provided hand-over-hand tactile guidance for correct hand posture. Participants were provided with verbal encouragement to maintain active engagement in the activity for the 20 min assigned to each category of activity. If they indicated feeling bored with the activity, they were allowed to switch to a different task within the same activity category. A 2–5 min rest period was provided between each activity category if they indicated feeling tired.

### Peripheral Nerve Somatosensory Stimulation

Peripheral nerve somatosensory stimulation was delivered bilaterally (Digitimer DS7AH, Digitimer Ltd., Welwyn Garden City, UK) with surface electrodes placed on the volar aspect of each wrist targeting the distribution of the median nerve (current parameters 10 Hz, 1 ms pulse duration, on/off duty cycle 500 ms/500 ms). The stimulation intensity was progressively increased to an intensity at which a muscle twitch could be observed in the thumb, and then decreased slightly below this level for the remainder of the session. Every 30 min, a study investigator reassessed the stimulation level, to ensure that there were no muscle contractions or habituation of the stimulation (measured by assuring that the individual indicated the perception of the intensity of the stimuli to be 4–6 on a 10-level visual analog scale). In the PNSS group, participants were discouraged from doing activities using their hands, but were allowed to watch movies or read.

### Conventional Exercise Training

Conventional exercise training consisted of a structured exercise protocol targeting strength (2 days/week) and endurance (3 days/week). The prescription of resistance load for strength training was performed based on submaximal repetitions using the following regression equation (41): 1-RM = wt/(0.522 + 0.419e^−^0.055 reps) where wt = load used in the exercise set in which >3 but <8 repetitions could be completed, reps = number of repetitions completed prior to fatigue. Participants were fitted to a wheelchair-accessible multi-station exercise device by a technician and performed the following exercises: chest press, shoulder press, dips, horizontal row, latissimus pull down, and biceps curls. In addition, resisted wrist flexion and extension exercises were performed with cuff weights. In each session, participants completed the following: 60% 1-RM × 5 repetitions, 75% 1-RM × 3 repetitions, and 90% 1-RM × as many repetitions as possible. The weight and number of repetitions completed during the third set of each exercise was used to calculate the new 1-RM for that exercise for the following week of training. For the endurance training, participants engaged in activities requiring hand grasp, and were allowed to choose between using a cycle ergometer (Monark Exercise AB, Vansbro, Sweden) or a virtual reality interface (XaviX; SSD Co Ltd., Kusatsu, Japan) where the participants could engage in games (e.g., tennis, baseball, and golf). Participants performed bouts of 30 min of endurance exercise at an intensity that was deemed tolerable and were allowed to rest for 2–5 min between bouts if they indicated feeling tired.

### Outcome Measures

#### Precision Grip and Power Grip Force Measurement

Precision grip (i.e., lateral prehension “key pinch” grip) strength and power grip (cylindrical grasp) strength were assessed with a handheld dynamometer (Microfet4; Hoggan Health Industries, West Jordan, UT, USA). While lateral prehension can serve both precision and power functions, it is among the most common grips for picking up small objects (42), and also represents a type of grip that is more frequently used by person with tetraplegia compared to tip-to-tip grip (43). During testing, the UE was stabilized on an adjustable height table in a standardized position with forearm resting, shoulder at 30°–40° flexion, adduction, and neutral rotation, elbow flexed to 90°, wrist in neutral. The participant was asked to pinch (using a lateral prehension grip) or squeeze (using a power grip) the dynamometer as hard as possible for 3 s. Three trials were performed at each test point, and the mean of these trials was calculated.

#### Sensory Measures

Semmes-Weinstein monofilament testing was used to measure the degree of sensitivity in the median nerve region (44, 45). This test includes five monofilaments ranging in diameter from 2.83 to 6.65 mm, and the test is scored based on the smallest monofilament the subject is able to detect in at least 50% of the trials. The region of the hand innervated by the median nerve was tested at the tip of the thumb, tip of the index finger, and base of the index finger. With the participant’s eyes closed, the smallest monofilament was used first. The monofilament was depressed until it bent and was removed after 1.5 s. The participant was instructed to respond verbally when a touch was perceived. If the individual did not respond, the next larger monofilament was used. Increasingly larger monofilaments were used until the participant responded to at least 5 out of 10 stimuli with the same monofilament. The monofilament diameter was coded on a 6-point scale wherein a lower score indicates poorer sensory perception, and a higher score indicates better sensory perception as follows: 5, 2.83 mm; 3, 4.31 mm; 4, 3.61 mm; 2, 4.56 mm; 1, 6.65 mm; 0, unable to detect the largest monofilament. The scores for the three median nerve innervated sites were averaged for a maximum sensory score of 5.

#### CSE Measures

Relationships between force production, sensory function, and CSE were assessed in a subset of nine participants. Testing was performed with participants seated in their wheelchair or padded examination chair with the upper extremities supported on a tray table. For the assessments of CSE, we tested the weaker UE unless the participant had no visible thenar muscle contraction on this side, in which case the stronger side was tested (*n* = 2). The skin overlying the abductor pollicis brevis (thenar muscle) was prepared, and surface electromyography (EMG) electrodes (Ag–AgCl, 10 mm) were adhered over the muscle belly. EMG signals were amplified, filtered (10 Hz–1 kHz), and sampled at 2 kHz (CED 1401, Cambridge Electronic Design, Cambridge, UK). We identified the level of EMG associated with maximum voluntary contraction of thenar muscles; at a target window of 10–15%, the maximum EMG value was constructed and displayed on a computer screen (46). Participants were asked to maintain their EMG at a quiet baseline during the resting CSE tests and to maintain their EMG within the target window during the active CSE testing.

To assess CSE, MEPs were elicited via TMS (Magstim 200, Magstim Company Ltd., Dyfed, UK) using a figure-eight coil (loop diameter, 70 cm). The coil was positioned tangential to scalp, handle directed posteriorly, and 45° away from midline. We identified the location that elicited the largest potential at the lowest stimulation intensity (ie, hot spot). At the hot spot, we identified the resting and active motor thresholds (RMT and AMT, respectively). RMT was defined as the lowest stimulator intensity at which a MEP of at least 50 µV could be evoked in at least 5 of 10 trials, and AMT was defined as the lowest stimulator intensity at which a MEP of at least 100 µV above background EMG could be evoked at least 5 of 10 trials. For all neurophysiologic outcome measures, peak-to-peak amplitudes were analyzed offline using Signal v5.0 (Cambridge Electronic Design, UK). Files were corrected for DC offset prior to analysis by determining the average amplitude found in the pre-stimulus period (−184 to −4 ms). Amplitude was then offset by the inverse of this value for all frames. In most cases, MEPs were measured in the 20–40 ms latency window after stimulus. If the MEP latency fell reliably outside of this interval, cursors were moved to ensure that the largest MEP peak wave was captured; however, the recording window remained 20 ms in duration. Qualifying MEPs were defined as having peak-to-peak amplitude of at least 50 µV in the “rest” condition, or an amplitude greater than 100 µV in the “active” condition after background motor activity (recorded in the pre-stimulus period) was removed. Frames were excluded from analysis if the stimulus artifact leaked into the physiological latency. These were identified via visual inspection and confirmed by consensus of three of the study team members.

An active recruitment curve was constructed beginning at stimulator intensity of 80% AMT and increasing in 5% increments up to maximum MEP amplitude, with five MEPs recorded at each stimulation intensity. To construct the recruitment curve, up to five qualifying MEPs at the same stimulus intensity were averaged and normalized to the maximum baseline MEP (MEP_max_). Values were plotted against the corresponding stimulator output (%Maximum Stimulator Output; MSO). The amplitude of the MEP at a stimulator intensity 120% of MT (MEP_120_) was identified, and slope of the recruitment curve (RC_slope_) between 100 and 140% of the AMT was calculated. The intensity range, as a percentage of AMT, over which MEPs could be evoked was identified (RC_range_). The area under the recruitment curve (RC_AUC_) was calculated pre- and post-intervention following published methods (47). Briefly, area was calculated using a cumulative trapezoid method, bounded by stimulation intensity and normalized MEP amplitude. Because recruitment curves were collected based on time period-dependent AMTs, participant pre- and post-intervention recruitment curves did not always share the same stimulation intensity bounds. However, comparison of within-subject pre- and post-intervention AUC requires consistent intensity bounds. To achieve this, when necessary MEPs at specific %MSO were estimated using a linear interpolation between the two surrounding measured MEP values.

#### Data Analysis

Descriptive statistics were calculated for participant characteristics and outcome measures. To assess within-group training effects, we used one-tailed paired sample *t*-tests, and to evaluate the meaningfulness of the changes, we calculated the standardized response mean (SRM) (48). We interpreted *p* < 0.05 as a statistically significant change and interpreted the clinical meaningfulness of the size of the effects based on the conventional cut-points of the following: trivial effect, <0.20; small effect, ≥0.20 < 0.50; moderate effect, ≥0.50 < 0.80; or large effect, ≥0.80 (49). To assess whether responsiveness was associated with baseline sensory function or was a function of baseline values of each measure, we calculated Pearson correlation coefficients to examine the relationship between baseline sensory function and the strength-related outcomes, and between baseline measures and their respective post-intervention values. Pearson correlation coefficients were also used to assess whether changes in grip strength (precision grip, power grip) and sensory scores were related to baseline CSE. Correlations between neurophys measures and strength measures were calculated using data from the same hand. This was the weaker hand for all but two subjects. In these two subjects no visible contraction was observed in the thenar muscles of the weaker hand, and therefore, the stronger hand was tested.

## Results

Of the 49 participants who entered the study, 37 completed the training and assessment sessions. One participant was withdrawn prior to the initial testing sessions when it was discovered that he was concurrently enrolled in another study. Eleven participants withdrew over the course of the 4-week intervention: five after deciding they could not adhere to the time commitment due to other obligations, four were dissatisfied with their group allocation, one had a family emergency, and one declined to offer a reason. Pre–post-intervention data were available for 37 participants (Table 1). There were no statistically significant differences in baseline measures among the three intervention groups, and the predominance of males in the study sample is consistent with the demographics of SCI (1).

**Table 1:** **Participant baseline characteristics**.

Characteristic		Full sample	FTP + PNSS	PNSS	CET
		*n* = 37	*n* = 14	*n* = 13	*n* = 10
Sex					
Men:women		30:7	12:2	12:1	6:4

Age (years)					
Mean (SD)		37.9 (14.6)	42.4 (13.5)	34.2 (16.4)	36.6 (13.2)

Time post-injury (years)					
Mean (SD)		8.5 (10.4)	13.7 (12.9)	6.5 (9.0)	4 (3.8)

Level of injury					
Median (range)		C6 (C4–8)	C6 (C5–8)	C6 (C4–7)	C6 (C5–7)

AIS classification		*A* = 1; *B* = 5; *C* = 29; *D* = 2	*A* = 0; *B* = 3; *C* = 11; *D* = 0	*A* = 1; *B* = 2; *C* = 9; *D* = 1	*A* = 0; *B* = 0; *C* = 9; *D* = 1

UEMS median (min–max)	Weak UE	13 (4–22)	10.5 (5–22)	13.0 (4–21)	14.0 (5–18)
	Strong UE	15 (8–25)	14.0 (8–25)	15.5 (8–23)	14.5 (9–24)

Precision grip (N), mean (SD)	Weak UE	10.72 (20.0)	14.23 (23.1)	5.78 (8.9)	12.01 (26.2)
	Strong UE	19.66 (26.7)	26.69 (32)	18.68 (27.1)	10.68 (15.1)

Power grip (N), mean (SD)	Weak UE	19.26 (35.1)	27.98 (46.3)	12.41 (19.6)	14.68 (32.9)
	Strong UE	23.13 (38.7)	32.74 (53.8)	18.90 (24.5)	14.06 (25.8)

Sensory mean (SD)	Weak UE	3.70 (1.4)	2.97 (1.6)	3.97 (1.4)	4.26 (1.0)
	Strong UE	3.84 (1.2)	3.2 (1.6)	3.95 (0.8)	4.50 (0.6)

*AIS, ASIA Impairment Scale; CET, conventional exercise training; FTP + PNSS, functional task practice plus peripheral nerve somatosensory stimulation; UE, upper extremity; UEMS, upper extremity motor score*.

**Figure 2:**
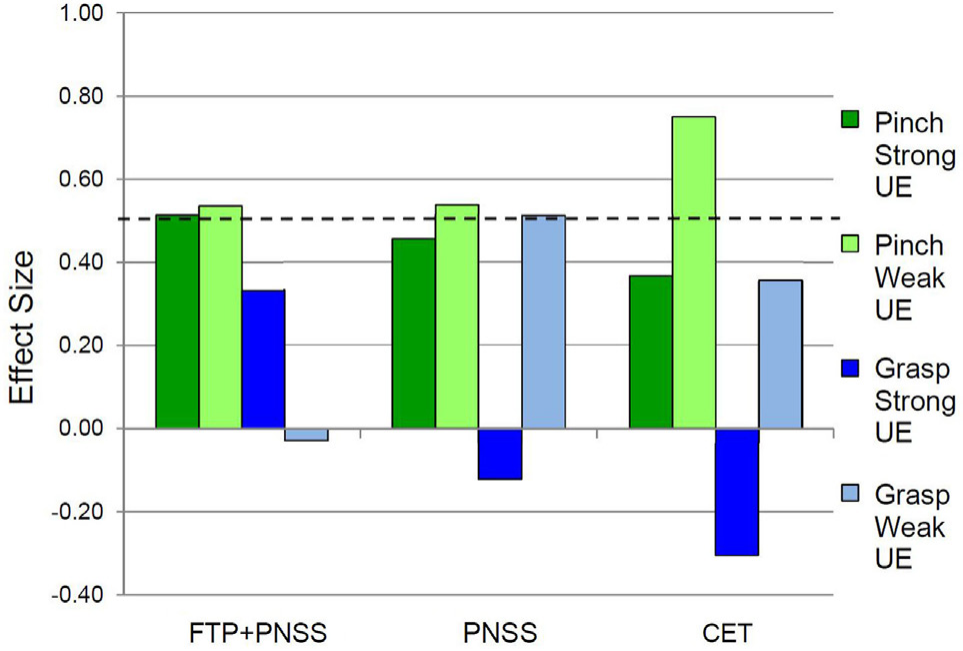
**Effect sizes for outcomes related to precision vs. power grip**. In the weak hand, the standardized response mean met the criterion of 0.5 effect size indicating meaningful change for Pinch (precision grip) and Grasp (power grip) for all three interventions. In the strong hand, the 0.5 criterion were met only in the FTP + PNSS group.

Following the intervention, the FTP + PNSS group had statistically significant improvements in precision grip strength in both the stronger and weaker hand (mean: 8.45N, *p* = 0.04, and 6.67N, *p* = 0.03, respectively) (Table 2). Note that percentage change is included to facilitate comparison with other publications that have reported change in terms of percentage. In both the PNSS and CET groups, there were significant improvements in precision grip strength of the weaker hand (Mean: 3.11N, *p* = 0.04, and 1.33N, *p* = 0.02, respectively), but not the stronger hand. All measures that met the criterion for statistically significant change had a SRM that met the criterion for moderate effect size (Figure 2). It is notable that the values reported herein do not include precision grip measures from 18 hands from which zero force could be recorded at the start of the study but from which measureable force values were obtained after training, since any change from 0 would represent an immense change and we believed this would distort the result. However in 14/18, precision grip strength increased to a measureable value following training. Statistically significant improvements in power grip strength and somatosensory function were not observed in any of the three groups. However, in the PNSS group the mean change in power grip of the weaker hand approached significance (mean: 3.83N, p = 0.07) and the SRM for met the criterion for a moderate effect.

**Table 2:** **Change by intervention group and correlation between baseline and post-intervention measures**.

Pre–post change	N	FTP + PNSS		PNSS		CET	
		14		13		10	
		Change mean (SD)	Baseline vs. change relationship *r*-value^b^	Change mean (SD)	Baseline vs. change relationship *r*-value^b^	Change mean (SD)	Baseline vs. change relationship *r*-value^b^
			
		% change	[*p*-value]	% change	[*p*-value]	% change	[*p*-value]
			
		[*p*-value^a^]		[*p*-value^a^]		[*p*-value^a^]	
Precision Grip (N)	Weak UE	6.67 (12.5)	0.451	3.11 (5.8)	−0.089	1.33 (1.8)	−0.011
		59.3%	[0.53]	105.9%	[0.39]	6.8%	[0.49]
		[0.03*]		[0.04*]		[0.02*]	
	Strong UE	8.45 (16.5)	0.237	2.22 (4.9)	−0.141	1.33 (3.6)	−0.024
		42.6%	[0.21]	15.7%	[0.323]	−2.4%	[0.45]
		[0.04*]		[0.07]		[0.14]	
Power Grip (N)	Weak UE	−1.56 (5.4)	−0.63	3.83 (7.5)	0.81	3.02 (8.5)	0.88
		−25.2%	[0.014]*	10.1%	[0.002]**	−26.8%	[0.005]**
		[0.17]		[0.07]		[0.19]	
	Strong UE	10.99 (33.1)	0.144	−1.42 (11.7)	−0.31	−1.96 (6.4)	−0.77
		30.2%	[0.327]	−8.45%	[0.19]	22.7%	[0.023]*
		[0.14]		[0.35]		[0.22]	
Sensory (score)	Weak UE	−0.03 (0.48)	−0.23	−0.08 (0.86)	−0.624	0.07 (0.44)	−0.695
		10.7%	[0.23]	8.08%	[0.011]*	4.4%	[0.013]*
		[0.42]		[0.37]		[0.32]	
	Strong UE	0.03 (0.77)	−0.396	−0.39 (0.97)	0.249	0.001 (0.47)	−0.044
		8.75%	[0.09]	−12%	[0.21]	0.04%	[0.45]
		[0.45]		[0.09]		[0.49]	

*^a^Paired t-test, one-tailed*.

*^b^Pearson r, one-tailed*.

Bold text indicates significant values: *p < 0.05, **p < 0.01.

*Note that percentage change in included to facilitate comparison with other publications that have reported change in terms of percentage*.

*CET, conventional exercise training; FTP + PNSS, functional task practice plus peripheral nerve somatosensory stimulation; UE, upper extremity*.

**Figure 3:**
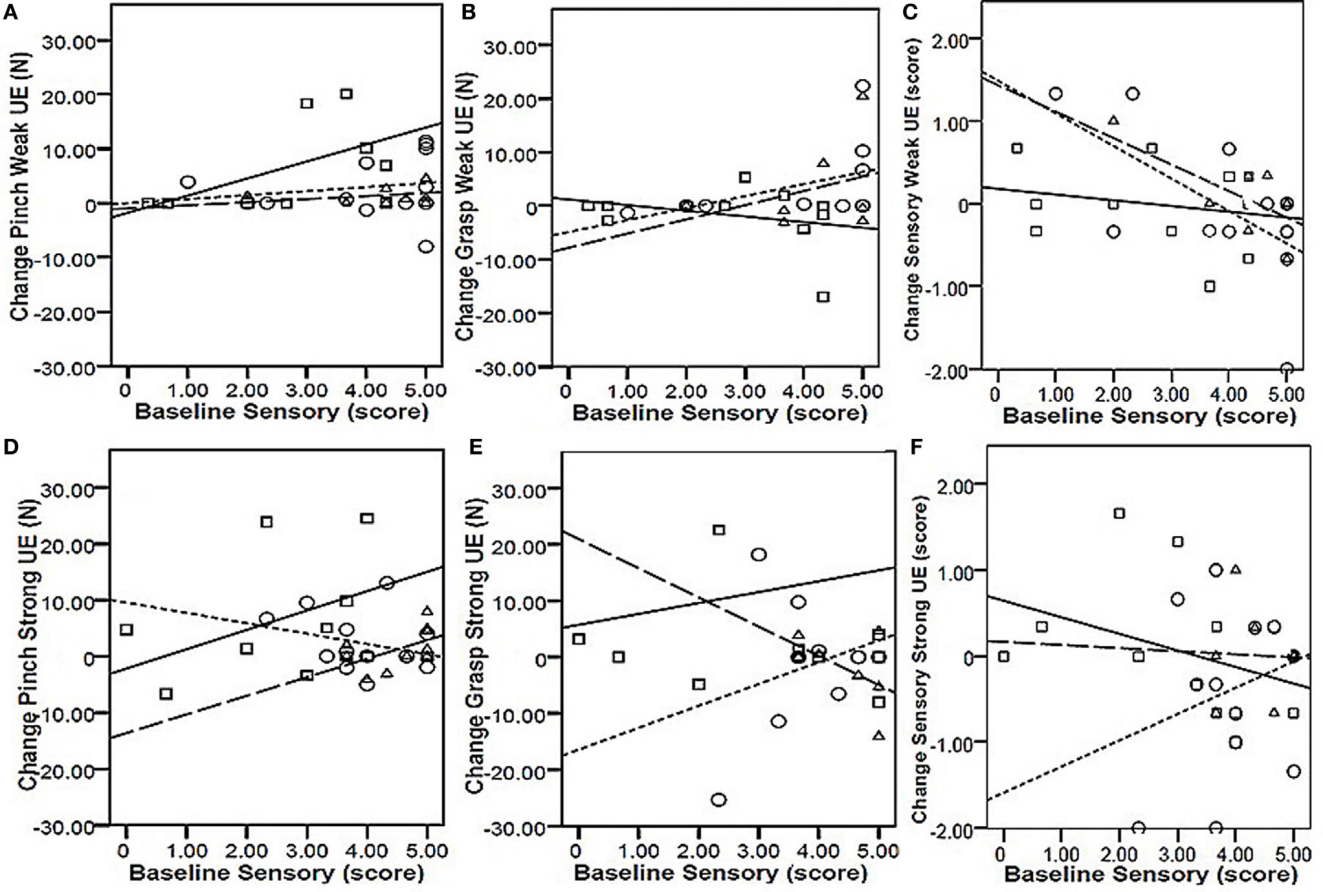
**Responsiveness as a function of baseline sensory scores**. Top row: change in Pinch [precision grip **(A)**], Grasp [power grip **(B)**], and Sensory function **(C)** in the weak hand as a function of baseline sensory scores of the weak hand. Bottom row: change in Pinch [precision grip **(D)**], Grasp [power grip **(E)**], and Sensory function **(F)** in the strong hand as a function of baseline sensory scores of the strong hand. Key (symbol, line): □ | = functional task practice plus peripheral nerve somatosensory stimulation (FTP + PNSS); ○ ⋮ = PNSS; Δ ¦ = conventional exercise training (CET). Pearson *r*-values were significant only for the relationship between baseline sensory scores and change in sensory function [Panel **(C)**] for both the PNSS (*r* = −0.624, *p* = 0.011) and CET group (*r* = −0.695, *p* = 0.013).

To determine whether baseline values of precision grip, power grip, and sensory function were predictive of responsiveness to change, we calculated the correlation between training-related change in these values as a function of the baseline values (Table 2). For all intervention groups, change in power grip force of the weaker hand was significantly correlated with baseline values. Similar significant relationships were observed for change in strong hand power grip as a function of baseline power grip in the CET group, and change in weak hand sensory score as a function of baseline sensory score in both the PNSS and CET groups. However, the clinical meaningfulness of these relationships is unclear given that changes in power grip force and change in sensory scores were small and not significant. Change in precision grip and power grip strength were only weakly related to baseline sensory function (*r* ≤ 0.5, *p* > 0.05 for all; Figure 3). However, the relationship between change in sensory score as a function of baseline sensory score was significant for both the PNSS (*r* = −0.624, *p* = 0.011) and CET groups (*r* = −0.695, *p* = 0.013) (Figure 3C), with the negative correlations indicating less change in participants who began with higher sensory scores.

Of the nine participants (4 FTP + PNSS, 3 PNSS, 2 CET) in whom the relationship between baseline CSE values and responsiveness to training were assessed, only two had thenar MEPs that could be evoked in the weak hand at rest. For that reason, CSE data related only to active contraction are reported. The relationship of interest was between measures of CSE and change in weak hand precision grip. Since all groups demonstrated change in precision grip force of the weaker hand, data were pooled across groups. Several measures of baseline CSE had moderate-to-strong correlations with change in precision grip (Table 3) of the weak hand. The CSE values related to AMT, MEP_120_, RC_range_, and RC_AUC_ were significantly correlated with change in weak hand precision grip strength. Since there was no significant change in sensory function, it follows that there was no significant relationship between baseline thenar CSE and change in somatosensory scores.

**Table 3:** **Relationships between baseline CSE and change in strength**.

Thenar CSE	Baseline CSE	Change in precision grip	Change in sensory

	Mean (SD)	Correlation^a^ [*p*-value]	Correlation^a^ [*p*-value]
Thenar AMT (%MSO)	47.89 (13.99)	−0.680 [0.022]*	−0.009 [0.491]
Thenar MEP_120_ (mV)	0.23 (0.1)	0.731 [0.031]*	−0.193 [0.339]
Thenar MEP_max_ (mV)	0.92 (0.5)	0.25 [0.26]	−0.429 [0.168]
Thenar RC_slope_ (×10^3^)	1.59 (4.32)	−0.37 [0.21]	−0.083 [0.416]
Thenar RC_range_ (%AMT)	117.78 (80.9)	0.800 [0.005]**	−0.193 [0.339]
Thenar RC_AUC_	48.66 (34.44)	0.648 [0.030]*	−0.199 [0.304]

*^a^Pearson r, one-tailed*.

Bold text indicates significant values: *p < 0.05, **p < 0.01.

*Measures of CSE were acquired from the cortex associated with the weaker hand (based on upper extremity motor score) in all but two subjects. In these subjects, no visible contraction was observed in the thenar muscles at baseline; therefore, the stronger hand was tested*.

*AMT, active motor threshold; CSE, corticospinal excitability; MEP120, amplitude of the motor-evoked potential at 120% of AMT; MEPmax, maximum motor-evoked potential; RCslope, slope of the recruitment curve between 100 and 140% AMT; RCrange, intensity range of the recruitment curve; RCAUC, area under the recruitment curve*.

## Discussion

The primary aim of this study was to examine the multisession effects of two different approaches to priming the corticospinal system (FTP + PNSS, PNSS) vs. CET on grip strength in persons with tetraplegia, and to understand the relationship between baseline measures and responsiveness to training. The findings indicate that the FTP + PNSS intervention was associated with significant improvements in precision grip strength of both the stronger and weaker hand. PNSS and CET were also associated with significant improvements, but only in the weaker hand. In all cases, the significant effects were associated with a moderate effect size (SRM > 0.5), suggesting a clinically meaningful effect (50). These results indicate that despite the fact that the FTP + PNSS intervention focused on task-related training rather than strengthening, this approach had a bilateral impact on precision grip. This is in contrast to CET which, despite a greater emphasis on strengthening, had an impact only on the precision grip of the weaker hand. Perhaps most interesting is that PNSS alone was associated with effects on precision grip that were similar to those exhibited by CET. Taken together, these results also support the concept that rehabilitation interventions aimed at priming CSE can improve hand strength even in persons with chronic tetraplegia. In fact, the FPT + PNSS group, which exhibited significant improvements in both hands, was the group with the greatest chronicity of SCI.

The finding that FTP + PNSS was associated with improvements in precision grip strength confirms the conclusions of prior smaller studies in persons with tetraplegia that have assessed the effects of this intervention on precision grip strength, but only in the weaker hand (12, 19). Imaging studies in neurologically healthy participants have shown that precision grip is associated with bihemispheric activation of numerous motor and sensory cortical regions, while power grip is associated primarily with activation of contralateral activation of the primary motor and sensory cortices (51). Since the FTP + PNSS group frequently used precision grip as a component of the FTP activities, and the CET group focused on activities requiring power grip, the differences in activity-related cortical activation may have contributed to the finding of bilateral increases in grip strength in the FPT + PNSS group, and only unilateral increases in grip strength of the CET group.

Somatosensory stimulation alone (i.e., PNSS) has previously been reported to improve precision grip strength of the weaker hand in persons with tetraplegia (12), presumably by increasing CSE (28, 29). While this is the first study to assess change in precision grip and power grip strength in persons with tetraplegia following CET, prior studies of persons with stroke have suggested that CET is associated with increased CSE (38). The results of the present study suggest that, assuming that increased CSE mediates the effects of precision grip strength of both PNSS and CET, the effect is sufficient to increase the strength only of the weaker hand, which would presumably have the greatest margin for improvement. However, it may be noteworthy that in the PNSS group, the change in precision grip strength of the stronger hand approached significance (*p* = 0.07) and the SRM met the criterion for a moderate effect size. Since FTP has been shown to increase CSE (25–27), it seems plausible that the combined effect of FTP+PNSS contributed to the finding that both the stronger and weaker hand benefited from this form of training.

An alternative explanation for the differences in outcome related to precision grip strength of the stronger hand for the FTP + PNSS group compared to the FTP and CET groups is that the FTP + PNSS group had a higher baseline strength in the stronger hand compared to the other two groups. However, the baseline strength of the stronger hand in the CET group (which did not increase) was equivalent to the baseline strength in the weaker hand in this group (which did increase). Likewise, the baseline strength of the weaker hand in the FTP group (which did increase) had the lowest baseline value, and for this reason, it seems unlikely that differences in outcomes for the FTP + PNSS vs. the FTP and CET groups are attributable to differences in baseline strength.

While the absolute values of change in precision grip strength may seem relatively small, there are many activities of daily living for which small changes in precision grip force may mean the difference between success or failure. For example, at the lower end of the spectrum of precision grip force requirements, Smaby et al. (6) estimated that approximately 5 N (approx. 1 lb) of grip force are required to insert a key into a lock, and 7 N (approx 1.5 lbs) are required to insert a card into an automatic teller machine (ATM). At the higher end of precision grip force requirements, approximately 15 N (approx. 3.5 lbs) of grip force are required to zip up a large zipper, while 18 N (approx. 4 lbs) of grip force are needed to pull out and 25 N (approx. 5.5 lbs) are needed to push in an electrical plug (6). Based on these values, one might surmise that an individual in the PNSS group who began with the mean precision grip strength in the weaker hand of just under 6 N (Table 1), and achieved the weak hand mean increase of 3 N (Table 2) would be able to use an ATM following of the intervention, when s/he may not have had this ability before. Likewise, a participant in the FTP + PNSS group who began with the mean precision grip strength of 14 N in the weaker hand may have struggled to plug in an electrical plug, but if this individual had achieved the mean increase of 6 N then s/he would have been able to accomplish this task following training.

None of the training approaches had significant effects on power grip strength. This result was rather unexpected as relates to the CET group, wherein many of the training activities required power grip to hold the handles of the multi-station exercise device or the ergometer. A possible explanation for the improvement in precision grip but not power grip strength may be differences in corticomotor drive in these two behaviors. Prior studies have indicated that the corticomotor system is more strongly activated during precision grip than it is during power grip (52). On this basis, it is plausible that the reverse also applies, wherein interventions that increase corticomotor activation have a larger effect on the ability to facilitate precision grip than the ability to facilitate power grip.

Given the reciprocal excitatory relationships between the motor and sensory cortices (10, 30, 53) and evidence that CSE is correlated with sensory function in persons with tetraplegia (9), the finding that none of the interventions was associated with change in sensory function was unexpected. This finding is in conflict with a prior report, which suggested that FTP + PNSS is associated with improvements in sensory function in persons with tetraplegia (12), as well as reports that PNSS is associated with improvements in sensory function in persons with multiple sclerosis (54). Given that PNSS directly activates the somatosensory cortex and that the movement associated with FTP would activate movement-related sensory afferents and thereby likewise activate the somatosensory cortex, this result was surprising. In contrast to the present study that employed bimanual training, in the prior study only the weaker hand was trained. It is possible that because both hands shared the practice tasks in the present study, this resulted in a smaller volume of movement in the weaker (and stronger) hand during training compared to the prior study, resulting in less overall afferent input to the cortex related to the weaker hand and therefore less influence on sensory perception.

Understanding the relationship between baseline measures and the magnitude of training-related change in a measure is valuable for predicting which individuals have the highest likelihood of obtaining a beneficial change associated with participation in training. For example, the published SCI rehabilitation literature contains some indications of the baseline measures that are associated with training-related change following locomotor training [for review, see Ref. (55)]. This information can assist in treatment planning, and decision making regarding the most appropriate interventions. However, in the present study, the only significant correlations observed between baseline measures and training-related change in the respective measures was in measures for which the training-related changes were not significant (power grip strength of the weaker hand and sensory scores; Table 2).

Since there were significant changes in weak hand precision grip strength across all intervention groups, an examination of the CSE measures associated with precision grip of the weak hand is worthwhile. The subset of participants from whom we obtained measures of CSE related to the thenar muscle of the weaker hand included participants from all training groups. There were significant relationships among several measures of baseline excitability and change in precision grip strength. Of the six baseline measures of CSE we examined, significant correlations with change in precision grip strength were observed in four of these. The negative correlation between thenar AMT and change in precision grip strength is consistent with the notion that those individuals from whom MEPs could be evoked with lower stimulus intensities at baseline showed the greatest responsiveness in terms of change in precision grip strength. Likewise, the positive relationships with MEP_120_, RC_range_, and RC_AUC_ suggest that improvements in precision grip strength were greatest in those individuals in whom it was possible to elicit MEPs with larger amplitude at a given stimulus intensity (MEP_120_), who had a larger range of stimulus intensities over which potentials could be evoked (RC_range_) and had MEPs of larger amplitude in that range (RC_AUC_).

The ability to exert compressive forces during precision grip is a coordinated activity that is highly dependent on extrinsic muscles with the assistance of intrinsic muscles; conversely, in power grip, the forces are exerted almost exclusively by the extrinsic muscles (56). All of the thenar muscles, with the exception of the adductor pollicis, are innervated by the median nerve (hence our targeting of the median nerve with PNSS) that is supplied by C6-T1 (sometimes also C5). Our inclusion criteria of visible contraction of the thenar muscles was intended to screen in participants who had some voluntary control of the intrinsic hand muscles. Since our focus was on priming of corticomotor excitability, this inclusion criteria was intended to capitalize on the direct corticomotor activation that subserves dexterous fingers control in primates [for review, see Ref. (57)]. Even passive movement has been shown to be associated with delayed increases in CSE (58). Therefore, FTP + PNSS engages bihemispheric cortical circuits (51) and has the potential to prime CSE in multiple ways, through the skill practice (FTP component) (25–27) and somatosensory stimulation (PNSS component) (28, 29), as well as through the natural afferent activation and somatosensory excitation that accompanies movement.

It is worthwhile to note that even in persons with SCIs classified as motor-complete, there may be partial innervation extending several segments below the neurological level of injury that is responsive to training. As a corollary, training-related increases in volitional control of lumbar-innervated muscles below the neurological level of injury are believed to underlie the improvement in walking function observed in association with locomotor training in an individual with complete thoracic SCI (59). There is every reason to believe that partially innervated muscles have the potential to respond to interventions that incorporate the concepts of motor priming, and thereby exhibit improved activation and associated contributions to function.

### Limitations

There are several limitations to this study. First due to participant withdrawals, the sample sizes were not equal across the three intervention groups. However, while the goal of randomization was to distribute the influence of confounding variables equally across groups, even with stratification there may be factors that contributed to the outcomes that were not accounted for by the stratification. Second, we did not attempt to quantify the training dose in terms of repetitions of the task in the FPT + PNSS or CET groups; such a measure may have been valuable for understanding differences in outcomes across subjects. However, undoubtedly, the number of repetitions was substantially higher than has been reported for inpatient rehabilitation wherein the median number of UE task repetitions for patients with tetraplegia is 42, and with 0 repetitions of hand-related activities (5). A third limitation of this study is that we assessed measures of CSE in a subset of participants. However, since precision grip force of the weaker hand changed in all groups, we believe that collapsing across groups and focusing on the CSE measures acquired from the thenar muscles, and their relationship to change in precision grip, allows meaningful conclusions to be drawn. Finally, we did not perform an objective assessment of spasticity, nor did we track the use of antispasmodic medications in our subjects. Prior studies have suggested that spasticity may influence response to training (15), and other studies have concluded that there may be differences in the way supraspinal circuits are engaged in persons with tetraplegia who are on a regimen of antispasmodic medications (9). It would be valuable for future studies to assess the influence of spasticity, as well as the effect of antispasmodic medications on response to UE training.

## Conclusion

Precision grip force is a necessary component of many tasks performed in daily life, and is typically impaired in people with cervical SCI. The findings of this study indicate that clinically accessible approaches to priming corticomotor activation, including functional task practice augmented by somatosensory stimulation and somatosensory stimulation alone are viable approaches to improving capacity for grip force production in the weaker hand. However, only functional task practice augmented by somatosensory stimulation had an effect on precision grip forces of the stronger hand. The amount of change observed may make a meaningful difference in the ability to perform activities of everyday life. Pre-training measures of CSE appear to be correlated with responsiveness to change in precision grip strength following intervention. Improvements in hand strength is possible even many years following injury.

## Author Contributions

EF-F designed the study. JG-O performed the subject recruitment and neurophysiologic assessments and assisted with development of the study protocol. JT processed the neurophysiologic data. JT, JG-O, and EF-F participated in the interpretation of the data. BP and JT performed the statistical analyses. EF-F, JG-O, and JT wrote the manuscript.

## Conflict of Interest Statement

The authors declare that the research was conducted in the absence of any commercial or financial relationships that could be construed as a potential conflict of interest. The reviewer RO and handling editor declared their shared affiliation, and the handling editor states that the process nevertheless met the standards of a fair and objective review.
